# Supplementary material for: Sexual dimorphic regulation of recombination by the synaptonemal complex in C. elegans
Source: eLife. 2023 Oct 5;12:e84538. doi: 10.7554/eLife.84538 (PMC10611432; doi:10.7554/eLife.84538)
Supplement: Figure 2—source data 2. [file elife-84538-fig2-data2.docx]

|  |  |  | **Pachytene nuclei #** | | |  |
| --- | --- | --- | --- | --- | --- | --- |
| **Genotype** | **Fluorescent protein** | **Sex** | **early** | **mid** | **late** | **# germlines** |
| WT | GFP::SYP-2 | hermaphrodite | 379 | 370 | 280 | 9 |
| WT | mCherry::SYP-3 | hermaphrodite | 294 | 490 | 411 | 14 |
| WT | GFP::SYP-2 | male | 227 | 240 | 220 | 12 |
| WT | mCherry::SYP-3 | male | 167 | 209 | 191 | 11 |
