# Supplementary material for: Sexual dimorphic regulation of recombination by the synaptonemal complex in C. elegans
Source: eLife. 2023 Oct 5;12:e84538. doi: 10.7554/eLife.84538 (PMC10611432; doi:10.7554/eLife.84538)
Supplement: Figure 3—source data 2. [file elife-84538-fig3-data2.docx]

|  |  |  | **Pachytene nuclei #** | | |  |
| --- | --- | --- | --- | --- | --- | --- |
| **Genotype** | **Fluorescent protein** | **Sex** | **early** | **mid** | **late** | **# germlines** |
| WT | GFP::SYP-2 | hermaphrodite | 379 | 370 | 280 | 9 |
| WT | mCherry::SYP-3 | hermaphrodite | 294 | 490 | 411 | 14 |
| *spo-11* | GFP::SYP-2 | hermaphrodite | 368 | 490 | 457 | 12 |
| *spo-11* | mCherry::SYP-3 | hermaphrodite | 214 | 362 | 340 | 9 |
| *cosa-1* | GFP::SYP-2 | hermaphrodite | 396 | 546 | 369 | 12 |
| *cosa-1* | mCherry::SYP-3 | hermaphrodite | 535 | 814 | 553 | 17 |
| WT | GFP::SYP-2 | male | 227 | 240 | 220 | 12 |
| WT | mCherry::SYP-3 | male | 167 | 209 | 191 | 11 |
| *spo-11* | GFP::SYP-2 | male | 178 | 204 | 155 | 9 |
| *spo-11* | mCherry::SYP-3 | male | 71 | 95 | 90 | 7 |
| *cosa-1* | GFP::SYP-2 | male | 131 | 186 | 157 | 7 |
| *cosa-1* | mCherry::SYP-3 | male | 123 | 176 | 167 | 8 |
