# Supplementary material for: Sexual dimorphic regulation of recombination by the synaptonemal complex in C. elegans
Source: eLife. 2023 Oct 5;12:e84538. doi: 10.7554/eLife.84538 (PMC10611432; doi:10.7554/eLife.84538)
Supplement: Figure 5—source data 2. [file elife-84538-fig5-data2.docx]

|  |  |  | **Pachytene nuclei #** | | |  |
| --- | --- | --- | --- | --- | --- | --- |
| **Genotype** | **Protein** | **Sex** | **early** | **mid** | **late** | **# germlines** |
| WT | RAD-51 | hermaphrodite | 375 | 412 | 339 | 8 |
| WT | MSH-5 | hermaphrodite | 465 | 618 | 519 | 11 |
| WT | COSA-1 | hermaphrodite | 533 | 459 | 384 | 9 |
| syp-2/+ | RAD-51 | hermaphrodite | 440 | 393 | 375 | 9 |
| syp-2/+ | MSH-5 | hermaphrodite | 479 | 440 | 384 | 10 |
| syp-2/+ | COSA-1 | hermaphrodite | 439 | 424 | 372 | 9 |
| syp-3/+ | RAD-51 | hermaphrodite | 382 | 336 | 282 | 9 |
| syp-3/+ | MSH-5 | hermaphrodite | 439 | 355 | 285 | 9 |
| syp-3/+ | COSA-1 | hermaphrodite | 386 | 359 | 329 | 9 |
| WT | RAD-51 | male | 471 | 420 | 476 | 10 |
| WT | MSH-5 | male | 301 | 285 | 363 | 9 |
| WT | COSA-1 | male | 303 | 269 | 301 | 12 |
| syp-2/+ | RAD-51 | male | 418 | 434 | 451 | 12 |
| syp-2/+ | MSH-5 | male | 268 | 303 | 343 | 9 |
| syp-2/+ | COSA-1 | male | 330 | 345 | 339 | 8 |
| syp-3/+ | RAD-51 | male | 376 | 296 | 383 | 10 |
| syp-3/+ | MSH-5 | male | 267 | 290 | 372 | 9 |
| syp-3/+ | COSA-1 | male | 290 | 359 | 272 | 7 |

**RAD-51, MSH-5, and COSA-1 n values for nuclei and germlines scored.**
