# Supplementary material for: Sexual dimorphic regulation of recombination by the synaptonemal complex in C. elegans
Source: eLife. 2023 Oct 5;12:e84538. doi: 10.7554/eLife.84538 (PMC10611432; doi:10.7554/eLife.84538)
Supplement: Figure 6—figure supplement 1—source data 1. [file elife-84538-fig6-figsupp1-data1.docx]

**Worm counts for Chromosome *X* SNP mapping recombination.**

|  |  | **Recombinant Intervals** | | | |  |  |
| --- | --- | --- | --- | --- | --- | --- | --- |
| **Sex** | **Genotype** | **A—B** | **B—C** | **C—D** | **D—E** | **Non-recombinant** | **Total worms** |
| **Oocytes** | Wild Type | 43 | 35 | 28 | 34 | 129 | 269 |
|  | *syp-2/+* | 22 | 22 | 18 | 29 | 172 | 263 |
|  | *syp-3/+* | 28 | 35 | 29 | 27 | 134 | 253 |
