# Supplementary material for: Sexual dimorphic regulation of recombination by the synaptonemal complex in C. elegans
Source: eLife. 2023 Oct 5;12:e84538. doi: 10.7554/eLife.84538 (PMC10611432; doi:10.7554/eLife.84538)
Supplement: Figure 7—source data 2. [file elife-84538-fig7-data2.docx]

|  |  |  | **Pachytene nuclei #** | | |  |
| --- | --- | --- | --- | --- | --- | --- |
| **Genotype** | **Fluorescent protein** | **Sex** | **early** | **mid** | **late** | **# germlines** |
| WT | GFP::SYP-2 | hermaphrodite | 379 | 370 | 280 | 9 |
| WT | mCherry::SYP-3 | hermaphrodite | 294 | 490 | 411 | 14 |
| *syp-2/+* | GFP::SYP-2 | hermaphrodite | 232 | 364 | 281 | 8 |
| *syp-2/+* | mCherry::SYP-3 | hermaphrodite | 222 | 296 | 186 | 8 |
| *syp-3/+* | GFP::SYP-2 | hermaphrodite | 301 | 323 | 255 | 10 |
| *syp-3/+* | mCherry::SYP-3 | hermaphrodite | 214 | 294 | 227 | 9 |
| WT | GFP::SYP-2 | male | 227 | 240 | 220 | 12 |
| WT | mCherry::SYP-3 | male | 167 | 209 | 191 | 11 |
| *syp-2/+* | GFP::SYP-2 | male | 144 | 144 | 124 | 9 |
| *syp-2/+* | mCherry::SYP-3 | male | 157 | 169 | 131 | 7 |
| *syp-3/+* | GFP::SYP-2 | male | 168 | 155 | 109 | 8 |
| *syp-3/+* | mCherry::SYP-3 | male | 163 | 173 | 137 | 7 |
