# Supplementary material for: Sexual dimorphic regulation of recombination by the synaptonemal complex in C. elegans
Source: eLife. 2023 Oct 5;12:e84538. doi: 10.7554/eLife.84538 (PMC10611432; doi:10.7554/eLife.84538)
Supplement: Figure 8—source data 2. [file elife-84538-fig8-data2.docx]

|  |  |  | **Pachytene nuclei #** | | |  |
| --- | --- | --- | --- | --- | --- | --- |
| **Genotype** | **Fluorescent protein** | **Sex** | **early** | **mid** | **late** | **# germlines** |
| WT | SYP-5::GFP | hermaphrodite | 174 | 416 | 367 | 8 |
| WT | SYP-6::GFP | hermaphrodite | 235 | 247 | 218 | 6 |
| *syp-2/+* | SYP-5::GFP | hermaphrodite | 206 | 429 | 353 | 9 |
| *syp-2/+* | SYP-6::GFP | hermaphrodite | 351 | 402 | 243 | 8 |
| *syp-3/+* | SYP-5::GFP | hermaphrodite | 165 | 338 | 260 | 9 |
| *syp-3/+* | SYP-6::GFP | hermaphrodite | 531 | 494 | 298 | 9 |
| WT | SYP-5::GFP | male | 165 | 186 | 173 | 10 |
| WT | SYP-6::GFP | male | 220 | 175 | 173 | 9 |
| *syp-2/+* | SYP-5::GFP | male | 165 | 132 | 101 | 8 |
| *syp-2/+* | SYP-6::GFP | male | 147 | 146 | 116 | 8 |
| *syp-3/+* | SYP-5::GFP | male | 102 | 116 | 97 | 7 |
| *syp-3/+* | SYP-6::GFP | male | 222 | 206 | 168 | 10 |
